# The osteogenic potential of human bone callus

**DOI:** 10.1038/srep36330

**Published:** 2016-10-31

**Authors:** Weiqi Han, Wei He, Wanlei Yang, Jianlei Li, Zhifan Yang, Xuanyuan Lu, An Qin, Yu Qian

**Affiliations:** 1 Department of Orthopaedics, Shaoxing People’s Hospital (Shaoxing Hospital of Zhejiang University), Shaoxing, Zhejiang 312000, PR China; 2 Department of Orthopaedics, Shanghai Key Laboratory of Orthopaedic Implant, Shanghai Ninth People’s Hospital, Shanghai Jiaotong University School of Medicine, Shanghai 200011, PR China

## Abstract

Bone callus, generated during fracture healing, is commonly discarded during surgical procedures. The aim of this study was to investigate the osteogenic potential of bone callus and its possible use as autograft material for patients needing bone grafts. Histology, immunohistochemistry, micro-computed tomography, and biomechanics were performed to examine osteogenic cells, osteoinductive factors, and the osteoconductive structure of bone callus. Alkaline phosphatase-positive osteoblasts, osteoinductive factors (including BMP2, FGF2, TGFB1, and IGF1), and a porous structure were found in bone callus. Early-stage callus (within 3 months after fracture) presented significantly improved osteogenic properties compared to medium- (3–9 months) and late-stage (longer than 9 months) callus. The results revealed that bone callus induced new bone formation in a nude mouse model. Early-stage callus showed better performance to medium- and late-stage callus in the induction of new bone formation at both 8 and 12 weeks. These findings indicated that bone callus, especially early-stage callus, possesses osteogenic potential and can potentially serve as an alternative source of material for bone grafts.

Bone callus is commonly observed at fracture sites. To ensure the accuracy of reduction and fixation during surgical procedures for fractures or nonunions, bone callus is commonly removed and discarded, especially in secondary surgery in nonunion patients^1^. Repairing a bone defect, healing of a nonunion, or surgery for the malunion of a fracture normally requires a bone graft using autologous bone harvested from the iliac crest^2^,^3^. However, performing such grafts is complicated by the additional surgical procedure required to obtain bone, and related donor site pain, infection, and unsightly scars^4^,^5^,^6^.

There is significant interest among orthopaedic surgeons to learn whether bone callus can be used as autograft material. Bone callus forms along with fracture healing, which is a process involving a complex interplay of cells, growth factors, and extracellular matrix^7^,^8^. At the cellular level, chondroblasts and osteoblasts are involved in callus formation^8^,^9^,^10^. At the molecular level, numerous osteoinductive growth factors are involved in callus formation, including bone morphogenetic proteins (BMPs)^9^,^11^,^12^,^13^, basic fibroblast growth factor (FGF2)^13^, transforming growth factor, beta 1 (TGFB1)^9^,^13^,^14^,^15^, vascular endothelial growth factor (VEGF)^9^,^13^,^16^, and insulin-like growth factor 1 (IGF1)^13^,^14^. In addition, bone callus tissue is histocompatible and non-immunogenic, reducing the likelihood of transmitting diseases when used as grafting material.

Nakase and colleagues reported that *in situ* grafting of excised bone callus, followed by external fixation, achieved a satisfactory therapeutic effect for the treatment of nonunions^17^. However, few studies have been conducted to systematically evaluate the osteogenic potential of bone callus. In the present study, we hypothesized that bone callus has osteogenic capability and can be used as bone graft material to induce new bone formation. To evaluate the osteogenic potential of bone callus, we assessed the roles of osteoblasts, osteoinductive growth factors, and the osteoconductive structures in bone callus by histological, immunohistochemistry, and micro-computed tomography (micro-CT) analyses. New bone formation induced by bone callus was determined in a nude mouse model.

## Results

### Density of bone callus

**Figure 1:**
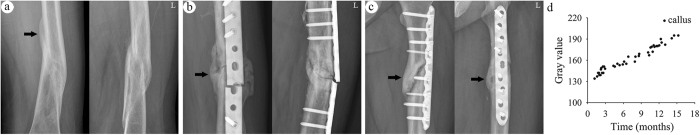
Bone callus formation at the fracture site (black arrows). (**a**) Anteroposterior and lateral radiographic views of malunion after plaster fixation in the EC group (**b**) Broken plates after internal fixation in the MC group (**c**) Nonunions after internal fixation in the LC group (**d**) Quantitative gray values of bone callus versus time of callus formation.

Bone calluses were observed at fracture sites by anteroposterior and lateral radiographs (Fig. 1a–c). The gray value of bone callus in the early-stage callus (EC) group was 143 ± 6. This value was significantly increased to 158 ± 6 in the medium-stage callus (MC) group and 182 ± 8 in the late-stage callus (LC) group (P < 0.001, Fig. 1d).

### Osteoblasts in bone callus

**Figure 2:**
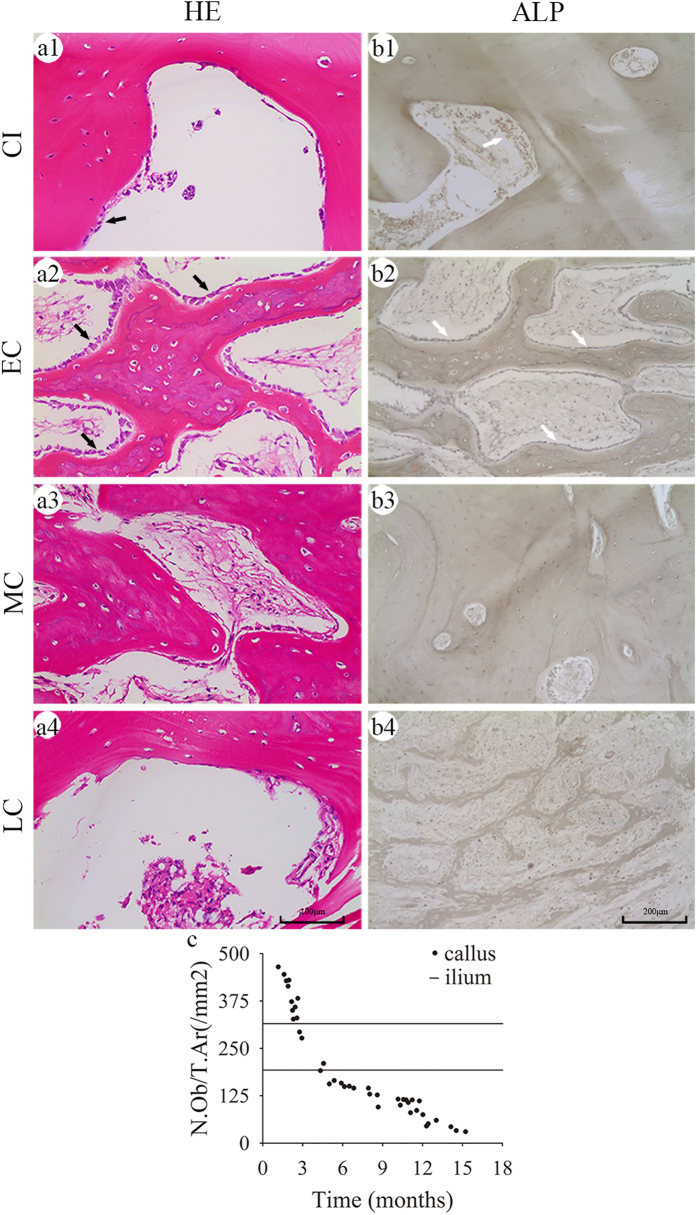
Histological evaluation of the number and activity of osteoblasts. **(a1–a4**) H&E staining revealed osteoblasts (black arrows) along the edge of the bone trabeculae (**b1–b4**) Active osteoblasts were stained for ALP (white arrows). Quantitative analyses showing the number of osteoblasts per unit of tissue area, N.Ob/T.Ar (/mm^2^) (**c**) in bone callus; the two black horizontal lines in each graph represent the upper and lower 95% confidence limits of the number and activity of osteoblasts in the CI.

Osteoblasts were identified according to their morphology and physiological localization in the bone callus, which lines the edge of the trabecular bone in a single layer (Fig. 2a). In the EC group, a large number of osteoblasts nearly covered the entire edge of the trabecular bone. Quantitative assessment showed that the number of osteoblasts in the EC group reached 375 ± 59/mm. In contrast, the numbers of osteoblasts in the MC and LC groups were significantly lower at 152 ± 30/mm and 80 ± 32/mm, respectively (Fig. 2c, Supplementary Table 1; P < 0.001). The number of osteoblasts in the control cancellous iliac (CI) group was 253 ± 85/mm. This value was lower than that in the EC group and higher than that in the MC and LC groups.

The activity of osteoblasts was examined by alkaline phosphatase (ALP) staining. Positive particles, stained brown-black, were localized in the osteoblasts (Fig. 2b). In the EC group, there were abundant positive particles in the osteoblasts. In contrast, positive particles in the MC and LC groups were relatively sparse and the staining was weaker than that in the CI group. Collectively, these results indicated that osteoblasts were plentiful in number and had robust activity in bone callus at 3 months post-fracture, which decreased later.

### Osteoinductive factors of bone callus

**Figure 3:**
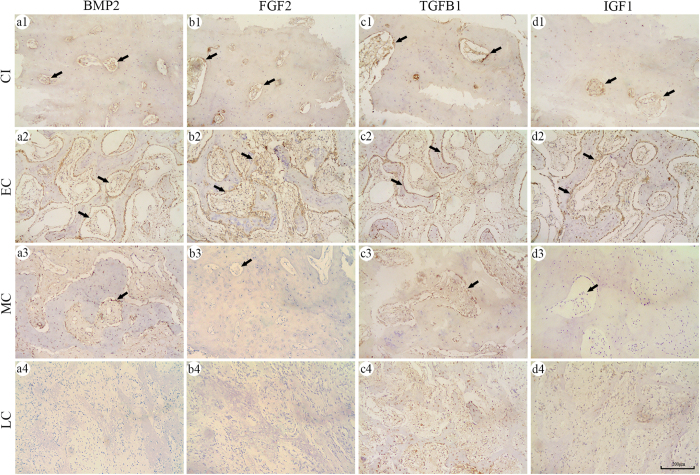
Expression of the osteoinductive factors BMP2, FGF2, TGFB1, and IGF1 in bone callus. Immunohistochemistry-positive areas for BMP2 (**a1–a4**), FGF2 (**b1–b4**), TGFB1 (**c1–c4**), and IGF1 (**d1–d4**) were observed as brown-black particles (black arrows).

Areas positively stained for the osteoinductive factors BMP2, FGF2, TGFB1, and IGF1 were observed in the bone callus at all stages, as well as in CI (Fig. 3a–d). In the EC group, there was a large area positive for BMP2 along the edge of trabecular bone. In contrast, positive areas in the MC and LC groups were relatively sparse and weaker than those in the CI group.

Similar to BMP2, FGF2, TGFB1, and IGF1 were expressed at higher levels in bone calluses at 3 months post-fracture and decreased thereafter. These results indicated that bone callus contained plentiful osteoinductive factors at 3 months post-fracture, which decreased remarkably thereafter.

### Microstructure of bone callus

**Figure 4:**
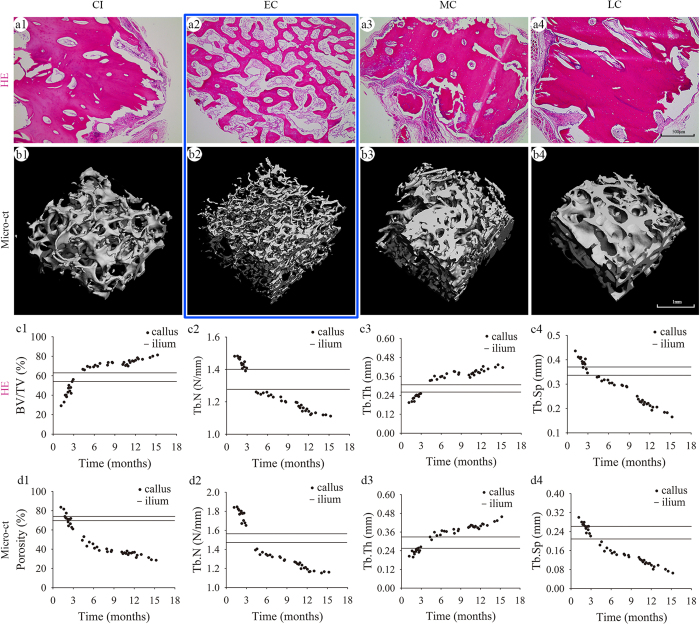
Microstructure of bone callus. H&E staining (**a1–a4**) and micro-CT scanning (**b1–b4**) showing internal connections and porous structure of the bone callus. (**c1–c4**) Two-dimensional analyses of BV/TV, Tb.N, Tb.Th, and Tb.Sp in H&E stained sections (**d1–d4**) Three-dimensional analysis of porosity, Tb.N, Tb.Th, and Tb.Sp observed by micro-CT; the two black horizontal lines in each graph represent the upper and lower 95% confidence interval of each parameter in the CI.

Histological examination and micro-CT scanning revealed the porous and internal cross-linked structure of bone callus. Histological examination showed regular morphology of trabecular or woven bone in the EC group, but mature lamellar bone in the MC and LC groups (Fig. 4a). Quantitative analyses showed that the bone volume over total volume (BV/TV) increased from 43.5% ± 7.7% in the EC group to 70.4% ± 2.6% in the MC group and 75.3% ± 3.4% in the LC group (Fig. 4c1). This trend indicated that the bony tissue in callus increased over time. In contrast, the trabecular number (Tb.N) and trabecular spacing (Tb.Sp) decreased with time, while the trabecular thickness (Tb.Th) exhibited a slight increase (Supplementary Table S2; P < 0.05).

Structural changes of bone callus were also evident by micro-CT scanning. Porosity in the EC group was 71.2% ± 6.9%, which was comparable to 71.5% ± 3.2% in the control CI group (Supplementary Table S3; P > 0.05). However, porosity decreased after 3 months post-fracture. Micro-CT analyses of Tb.N, Tb.Th, and Tb.Sp showed results similar to the histological examinations.

### Biomechanical features of bone callus

**Figure 5:**
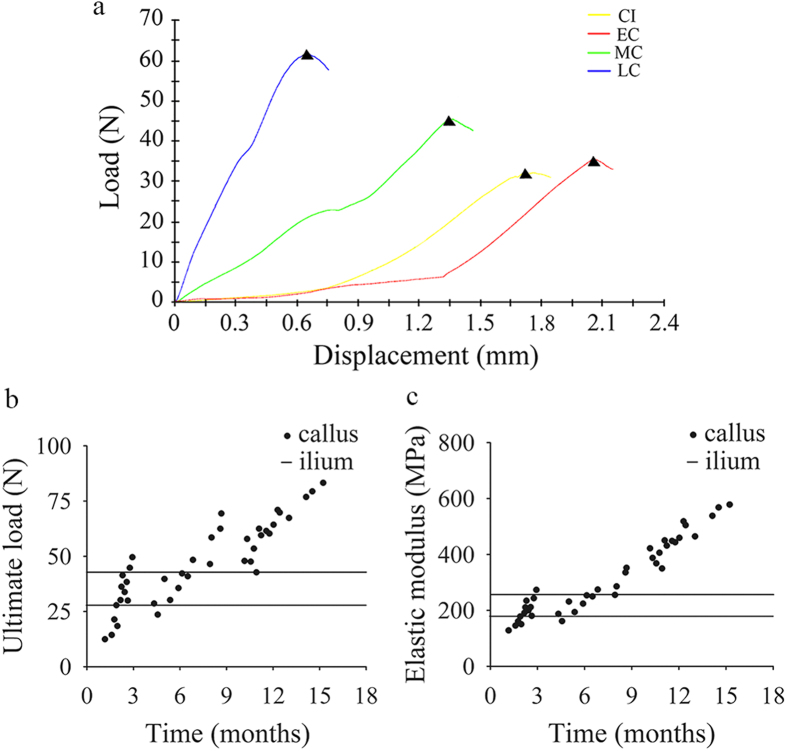
Bone biomechanics. (**a**) Representative load-displacement curves of bone callus obtained in the compression biomechanics test; ultimate load (**b**) and elastic modulus (**c**) of bone callus.

Typical load–displacement curves of bone callus and ilium are shown in Fig. 5a. The ultimate load of bone calluses increased from 30.6 ± 11.6 N in the EC group to 43.8 ± 14.0 N and 62.8 ± 11.6 N in the MC and LC groups, respectively. The ultimate load was 36.4 ± 10.5 N in the control CI group (Fig. 5b). No significant difference was observed in the elastic modulus between the EC (193 ± 42 MPa) and CI (224 ± 50 MPa; Supplementary Table S4; P = 0.111) groups. In contrast, the elastic modulus of the LC group (459 ± 68 MPa) was significantly higher than that in the CI group (Fig. 5c, Supplementary Table S4; P < 0.001). These results indicated that the compression biomechanical performance of the EC group was similar to that of the CI group, whereas the ultimate load and elastic modulus increased in the LC group.

### New bone formation induced by bone callus in nude mice

**Figure 6:**
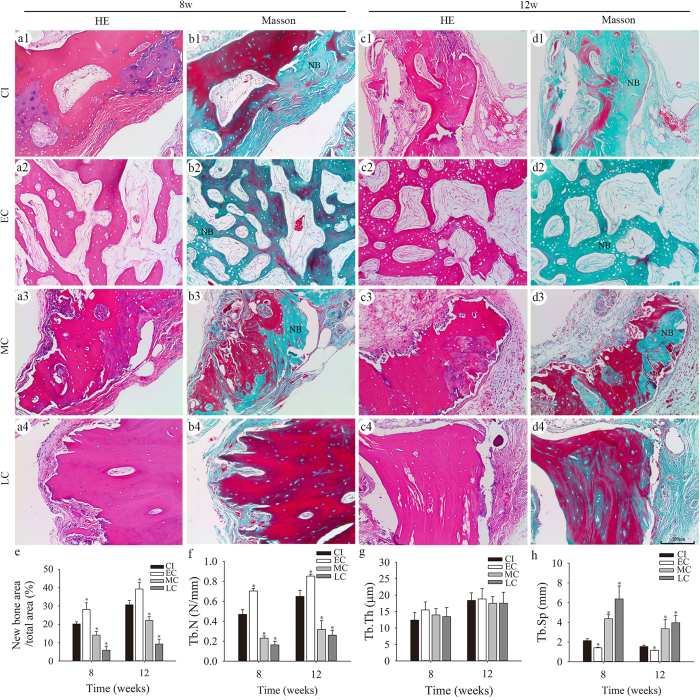
New bone formation induced by bone callus in nude mice as assessed by histomorphometry. A trabecula-like pattern of new bone formation was detected by H&E staining at 8 (**a1–a4**) and 12 (**c1–c4**) weeks post-implantation. New bone (NB) area stained green in Masson staining at 8 (**b1–b4**) and 12 (**d1–d4**) weeks after implantation; quantitative analyses of NB area (**e**), Tb.N (**f**), Tb.Th (**g**), and Tb.Sp (**h**), based on Masson staining; *P < 0.05 versus the CI group by one-way analysis of variance and Least Significance Difference (LSD) test post hoc.

The induction of new bone formation was observed using bone callus and ilium in serial sections stained with hematoxylin and eosin (H&E) or Masson’s trichrome, as well as by micro-CT scanning. H&E staining revealed plentiful osteoid and irregular trabecular bone formation in the EC group 8 weeks after implantation. In contrast, only fibrous tissue and sparse osteoid formation were evident in the CI group. There was less osteoid formation in the MC and LC groups than in the EC group (Fig. 6a). After 12 weeks, there was plentiful regular trabecular bone and medullary cavity formation in the EC group, and substantial osteoid and woven bone formation in the CI group. However, fewer woven bone formations were observed in the MC and LC groups (Fig. 6c). Similar results were observed with Masson staining; new bone formation was shown in green and existing bone in dark red (Fig. 6b,d). Quantitative analyses based on Masson staining revealed that the newly-formed bone area of the EC group was 28.3% ± 2.9% of the total area at 8 weeks and 39.5% ± 3.4% of the total area at 12 weeks after implantation. These values were significantly higher (P < 0.05) than those of the CI group at 8 (20.0% ± 1.8%) and 12 (30.8% ± 3.7%) weeks. The amounts of newly formed bone in the MC and LC groups were lower than those in the CI group at 8 and 12 weeks (Fig. 6e, Supplementary Tables S5 and S6; P < 0.05).

The Tb.N of new bone in the EC group at 8 and 12 weeks were significantly higher (P < 0.05) than those in the CI group, whereas these parameters in the MC and LC groups were lower than in the CI group. The values for Tb.Sp of new bone in the EC group at 8 and 12 weeks were significantly lower (P < 0.05) than those of the CI group, whereas these parameters were higher in the MC and LC groups than in the CI group. There were no significant differences in the Tb. Th of new bone among the four groups at 8 and 12 weeks (Fig. 6f–h, Supplementary Tables S5 and S6; P > 0.05).

**Figure 7:**
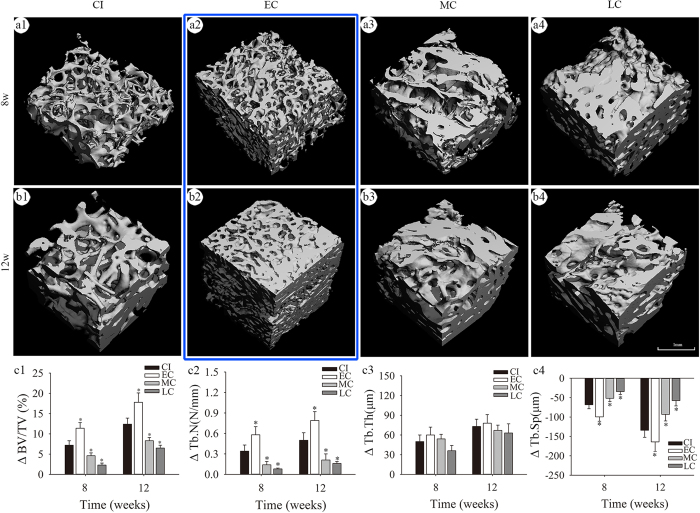
New bone formation induced by bone callus in nude mice assessed by micro-CT. Images showing three-dimensional reconstructions of regions of interest (3 mm × 3 mm × 3 mm each) in implants at 8 (**a1–a4**) and 12 (**b1–b4**) weeks after implantation; the ΔBV/TV (**c1**), ΔTb.N (**c2**), ΔTb.Th (**c3**), and ΔTb.Sp (**c4**) values between the parameters at 8 or 12 weeks post-implantation and these parameters before implantation; *P < 0.05 versus the CI group using one-way analysis of variance and LSD test post hoc.

The histomorphometric findings were confirmed by micro-CT scanning (Fig. 7). The ΔBV/TV, ΔTb.N, and ΔTb.Sp of new bone in the EC group were significantly higher than those of the CI group at 8 and 12 weeks (Fig. 7c1,c2 and c4; P < 0.05). In contrast, these parameters in the MC and LC groups were lower than the CI group at 8 and 12 weeks (P < 0.05). There were no significant differences in the ΔTb. Th of new bone among the four groups at 8 and 12 weeks (Fig. 7c3, Supplementary Tables S7 and S8; P > 0.05). Collectively, the osteogenic potential of bone callus was evidenced by the induction of new bone formation in nude mice. Furthermore, the osteogenic potential of bone callus within 3 months was greater than with the CI group.

## Discussion

Bone callus, as autologous tissue, has potential applications in bone grafting. The present study demonstrated the osteogenic capability of bone callus. Our study showed that bone callus had osteogenic potential in terms of osteoblast formation, osteoinductive factors, and an osteoconductive structure. Notably, the osteogenic capability of callus within 3 months was superior to the cancellous bone. The osteogenic capability of bone callus was also confirmed by the formation of new bone in nude mice. Together, these results indicated bone callus as an alternative source of bone graft material, and EC may be an extremely promising material for bone grafts.

With the formation of osteoblasts, osteogenic growth factors, and an osteoconductive structure (the key elements of osteogenesis), bone callus was shown to have significant osteogenic potential. However, the formation of both osteoblasts and osteogenic growth factors decreased with time, strongly indicating a limit to the osteogenic capability of bone callus. We believe that this downward trend is related to fracture healing and bone callus formation. When fracture occurs, the local injury stimulates chemotaxis and growth factor release, resulting in the rapid formation of numerous osteoblasts^18^. Osteogenic growth factors, mainly secreted by osteoblasts^19^, also reach peak concentrations at early time points^20^,^21^,^22^. As osteoblasts and osteogenic factors peak, callus formation and bone healing is initiated^23^,^24^,^25^. Specifically, once fracture occurs, mesenchymal stem cells are recruited to the wound site and differentiate into osteoblasts, which are responsible for secreting growth factors to induce osteogenic differentiation^26^. This positive feedback prompts the rapid growth of osteoblasts and high expression of growth factors^27^. This was evidenced by finding higher numbers of osteoblasts and greater amounts of osteogenic growth factors in the EC group than in the CI group.

As bone callus formation progresses, osteoblasts differentiate into osteocytes, undergo apoptosis, or convert to bone-lining cells (inactive osteoblasts), thereby decreasing the number of active osteoblasts^28^,^29^. As a result, the expression of osteogenic growth factors decreases with time. A sharp decline in the production of osteoblasts and growth factors was observed 3 months post-fracture. Once the porous bone callus is formed, fracture healing moves into a remodelling phase. During this phase, bony structural changes occur, including pore size, porosity, and the number and thickness of trabecular bone. However, the amount of bone does not increase in this phase as it does during the prior phase. Thus, the number of active osteoblasts and the expression of growth factors decrease sharply to a low maintenance level^28^,^29^. This event indicates that the rapid rebuilding of the fracture is complete and that healing has transitioned into a new stage of remodelling.

As a potential graft material, bone callus can be harvested from fracture sites, avoiding the donor-site complications of harvesting a traditional autologous bone graft, such as pain, bleeding, infection, and soft-tissue injury^30^,^31^. As an autologous tissue, bone callus has excellent histocompatibility, averting the limitation of immunological rejection and the risks of pathophoresis^32^,^33^ that can occur with allogeneic bone grafts. Moreover, bone callus simultaneously provides osteoblasts, osteoinductive factors, and an osteoconductive structure, making it an almost perfect osteogenic material with mechanical properties that are better than tissue-engineered bone. EC exhibits excellent osteogenic potential, but is relatively rare and available in only small quantities. MC and LC are much more common, but are limited by inferior osteogenic potentials. Therefore, bone callus may serve primarily an alternative or complementary bone graft material when bone grafting is required, such as in the following indications: (1) open reduction and internal fixation for long bone fracture after failure of conservative treatment; (2) open reduction and internal fixation for long bone fracture after temporary external fixation; (3) failure of internal fixation, including loosened or broken plates or screws, bent or broken intramedullary nails, and fracture angulation and aversion abnormalities, and (4) hypertrophic nonunion.

There were several limitations in this study. CI was used as the sole control for bone callus, and bone graft alternatives such as allogeneic bone and inorganic materials were not included. To demonstrate the internal structure of the callus, HE staining and micro-CT were performed (Fig. 4). Although obtained by two different methodologies (2D and 3D), the data from HE staining and micro-CT demonstrated a similar trend over time. To demonstrate the osteogenesis activity of bone callus in nude mice, Masson staining and micro-CT were performed (Figs 6 and 7). The data of Fig. 6 are based on the green area in Masson staining, which indicates the early stage of new bone formation. As described by Ren *et al*., new bone formation is stained green and part of the degenerative bone matrix is stained red in Masson staining^34^. The possible reason for this is that the collagen matrix degenerates gradually in the process of endochondral ossification, and degenerative collagen has a more regular, dense, and crosslinked structure, which may change the electronic charge characteristics of collagen^35^. The data in Fig. 7 show the differences in the bone callus matrix pre- and post-implantation, which may reflect the net new bone formation induced by the bone callus and resorption of callus tissue. Moreover, bone calluses harvested from different fracture sites might decrease the accuracy of the results. Bone callus samples were obtained from non-uniform sources, i.e. patients with failure of conservative treatment or internal fixation within 9 months, or those with hypertrophic nonunion after 9 months. In addition, fractures complicated by metabolic disorders were excluded from this study. This may have also led to some degree of bias on the results in this study. For instance, plate loosening may lead to a much more reactive callus than atrophic nonunion. It should be noted that bone calluses are not present in every case of nonunion, e.g. atrophic nonunion, which requires bone grafting, normally does not result in the generation of bone callus at the fracture site. The volume of the calluses harvested during the surgical procedures depended on numerous factors. The purpose of callus removal is improved reduction of the fracture and placement of plates. Since the implantation of calluses at the fracture sites was not addressed in this study, the surgeon did not remove the entire callus at the fracture sites. However, the volume of the callus is important for clinical translation of our findings, and we aim to investigate this aspect in future studies. Further investigation of the application of bone callus to bone defects and fracture nonunions in animal models and clinical settings is required to realize the clinical potential of this material.

## Methods

### Study design

To study the osteogenic potential of bone callus at different stages of callus formation, samples harvested from patients were divided into three groups: the EC group (within 3 months), the MC group (from 3 to 9 months), and the LC group (more than 9 months). CI was chosen as the control bone graft material.

To investigate osteogenesis of bone callus, the callus was stained with H&E, and Gomori for ALP. To evaluate the osteoinductivity of bone callus, samples were assessed by immunohistochemistry for the BMP2, FGF2, TGFB1, VEGF, and IGF1 proteins. To evaluate the osteoconductivity of bone callus, samples were analysed by H&E staining and micro-CT scanning. To assess new bone formation, human bone callus was implanted subcutaneously in nude mice. The formation of new bone was detected by H&E staining, Masson staining, and micro-CT scanning.

### Collection of bone callus and CI tissues

The project was approved by the Ethics Committee of Shaoxing People’s Hospital (No. 080) and the protocol was carried out in accordance with approved guidelines. Preoperative informed consent was obtained from each patient. From January 2010 to June 2014, bone callus samples were obtained from patients undergoing surgical treatment in the Department of Orthopedics of Shaoxing People’s Hospital. EC was collected from patients who required surgical treatment for failure of skeletal traction in four cases, fracture angulation or displacement after conservative treatment with plaster in the primary treatment in five cases, and after external fixation temporary for open fractures with severe soft tissue injury in two cases and multiple trauma in one case. MC was collected from secondary surgeries after loosened or broken plates or screws in eight cases, and bent or broken intramedullary nails in four cases. LC was collected from secondary surgeries for hypertrophic nonunion in 16 cases. CI was collected from patients who needed self-ilium grafts during the surgery. These patients exhibited healing post-surgery during the follow-up period.

Inclusion criteria were as follows: (1) surgeries after failure of conservative treatment or external fixation were applied temporarily before open reduction and plate fixation for long bone fractures; (2) secondary surgeries after failure of internal fixation, including loosened or broken plates or screws, bent or broken intramedullary nails, and fracture angulation and aversion abnormalities; and (3) secondary surgeries for hypertrophic nonunion. Exclusion criteria were as follows: (1) fracture complicated with microbial infection; (2) fracture complicated with brain injury; (3) bone tumours; (4) systemic bone-related diseases; and (5) patients treated with hormones, steroids, vitamin D, or calcium. The general characteristics of the patients are presented in Table 1.

### Radiological measurements

Radiographs of the anteroposterior and lateral views of the fracture sites were taken before bone callus samples were harvested. The default acquisition parameters included a source image distance of 120 cm, 55 kV, 5 mAs, and no added tube filtration. According to the scan protocol, image post-processing was performed at a set window width of 3845 and window level of 2031. The gray value of bone callus was judged with a Picture Archiving and Communication System (RADinfo Inc., Hangzhou, Zhejiang, China), using Image-Pro Plus (IPP) 6.0 software (Media Cybernetics Inc., Bethesda, MD, USA).

### Histological and immunohistochemical analyses

Bone samples harvested from patients, and implants from mice, were fixed and decalcified in 10% ethylenediaminetetraacetic acid at pH 7.4 for 1 month. The maximum cross-sectional area of the calluses was selected as the embedding surface, which was parallel to the bottom of the embedding box. Thus, the maximum cross-sectional area of the calluses could be included in the sections. To ensure that the mid part of the regenerating callus was included in histological sections, rough trimming with sectioning was performed until the maximum cross-sectional area of callus tissue was nearly exposed in the sections, and serial sectioning (5 μm) was then started. In total, 60 sections per sample were collected. The sections were stained with H&E, Gomori, immunohistochemistry, and Masson’s trichrome. The number of osteoblasts was quantitated in five nonconsecutive sections per sample after H&E staining. Values for the BV/TV, Tb.Th, Tb.N, and Tb.Sp were calculated by H&E staining, as described by Zhang *et al*.^36^. In addition, the area of new bone formation induced by callus in nude mice was measured in sections using IPP software. Immunohistochemistry was performed with a mouse anti-BMP2 monoclonal antibody (#ab6285; Abcam, Cambridge, UK), and rabbit polyclonal antibodies against FGF2 (#ab16828), TGFB1 (#ab66043), and IGF1 (#ab9572).

### Micro-CT scanning

The structure of bone callus harvested from patients, and the implants harvested from mice, were analysed using a μCT100 micro-CT scanner (Scanco Medical, Bruttisellen, Switzerland). Scanning was performed at 70 kV and 200 μA, with a resolution of 10.0 μm per pixel. Image reconstruction software (μCT V6.1) and three-dimensional model visualization software (μCT Ray V4.0) were used for further analyses. The volume of interest (VOI) was drawn in 3 mm × 3 mm × 3 mm (27 mm^3^) cubes at the centre of the calluses. Porosity, Tb.Th, Tb.N, and Tb.Sp were then measured using data analysis software (μCT Evaluation Program V6.5). The precise borders of the implants were poorly defined at 8 weeks and 12 weeks post-implantation. To study new bone formation within the implants, the region of interest was limited to 3 mm × 3 mm × 3 mm at the centre of the implants. ΔBV/TV, ΔTb.Th, ΔTb.N, and ΔTb.Sp were calculated as the differences between the parameters post- (8 or 12 weeks) and pre-implantation.

### Biomechanics testing

The compression test of bone callus from patients was performed using an Instron 3366 dynamic mechanical analyser (Instron; Norwood, MA, USA). Samples were cut into 5 mm × 5 mm × 5 mm cubes, fixed with bone cement, and exposed to a cross-load at 2 mm/min, using a 2-mm diameter Kirschner wire. The displacement and applied force were recorded during the test, and the ultimate load and elastic modulus were calculated from the stress-strain curve.

### Animal model

All procedures involving animals were approved by the Ethics Committee of Shaoxing People’s Hospital (No. 079) and conformed to its guidelines. Six-week-old male nude mice (body weights 20–25 g; Animal Resources Centre, Shanghai, China) were assigned randomly into the EC group, MC group, LC group, and CI groups (4 mice per group) for each time point. Anaesthesia was induced with intraperitoneal pentobarbital (50 mg/kg). All surgeries were performed under aseptic conditions. Two longitudinal incisions (approximately 1 cm) were made over the iliac crest and 5 mm bilaterally from the posterior midline. The volume and shape of the calluses or ilium were highly variable among the patients. Suitable samples (large enough) were selected for subcutaneous implantation and biomechanics testing, and the remaining samples were used in histological analysis, immunohistochemistry and micro-CT scanning. Two bone callus or ilium samples (5 mm × 5 mm × 5 mm each) were then implanted subcutaneously into each mouse. The incisions were closed with sutures, and mice were subsequently scanned with CT along the axial plane. Four animals per group per time point were used, and two bone implant samples were implanted subcutaneously into each animal. Thus, eight samples were implanted per group per time point.

### Statistical analyses

Data are expressed as means ± standard deviation, and statistical analyses were performed using SPSS software, version 16.0 (IBM, Chicago, IL, USA). Student-Newman-Keuls multiple comparisons test was performed to analyse donor characteristics. Statistical analyses of radiographic score, histologic examination, micro-CT results, and biomechanical testing were performed by one-way analysis of variance and Least Significance Difference (LSD) test post hoc. P values of < 0.05 were considered statistically significant.

## Additional Information

**How to cite this article**: Han, W. *et al*. The osteogenic potential of human bone callus. *Sci. Rep.* **6**, 36330; doi: 10.1038/srep36330 (2016).

**Publisher’s note:** Springer Nature remains neutral with regard to jurisdictional claims in published maps and institutional affiliations.

## Supplementary Material

Supplementary Information

## Figures and Tables

**Table 1:** Donor characteristics.

Group	Cases, n	Age (years)	Sex (cases, n)		Drawn parts (cases, n)		
			Males	Females	Femur	Tibia	Humerus
CI	11	45.7 ± 8.4	5	6	—	—	—
EC	13	45.5 ± 7.7	8	5	6	4	3
MC	12	46.3 ± 5.9	7	5	5	5	2
LC	16	45.6 ± 6.4	9	7	5	7	4
P value	—	0.991	0.895		0.941		

Group differences were analysed by Student-Newman-Keuls multiple comparisons test.
