# Supplementary material for: The osteogenic potential of human bone callus
Source: Sci Rep. 2016 Oct 31;6:36330. doi: 10.1038/srep36330 (PMC5087090; doi:10.1038/srep36330)
Supplement: Supplementary Information [file srep36330-s1.doc]

**Supplementary Information**

**The osteogenic potential of human** **bone callus**

Weiqi Han^a,1^, Wei He^a,1^, Wanlei Yang^a^, Jianlei Li^a^, Zhifan Yang^a^, Xuanyuan Lu^a^, An Qin^b^ & Yu Qian^a,*^

^a^Department of Orthopaedics, Shaoxing People’s Hospital (Shaoxing Hospital of Zhejiang University), Shaoxing, Zhejiang 312000, PR China

^b^Department of Orthopaedics, Shanghai Key Laboratory of Orthopaedic Implant, Shanghai Ninth People’s Hospital, Shanghai Jiaotong University School of Medicine, Shanghai 200011, PR China.


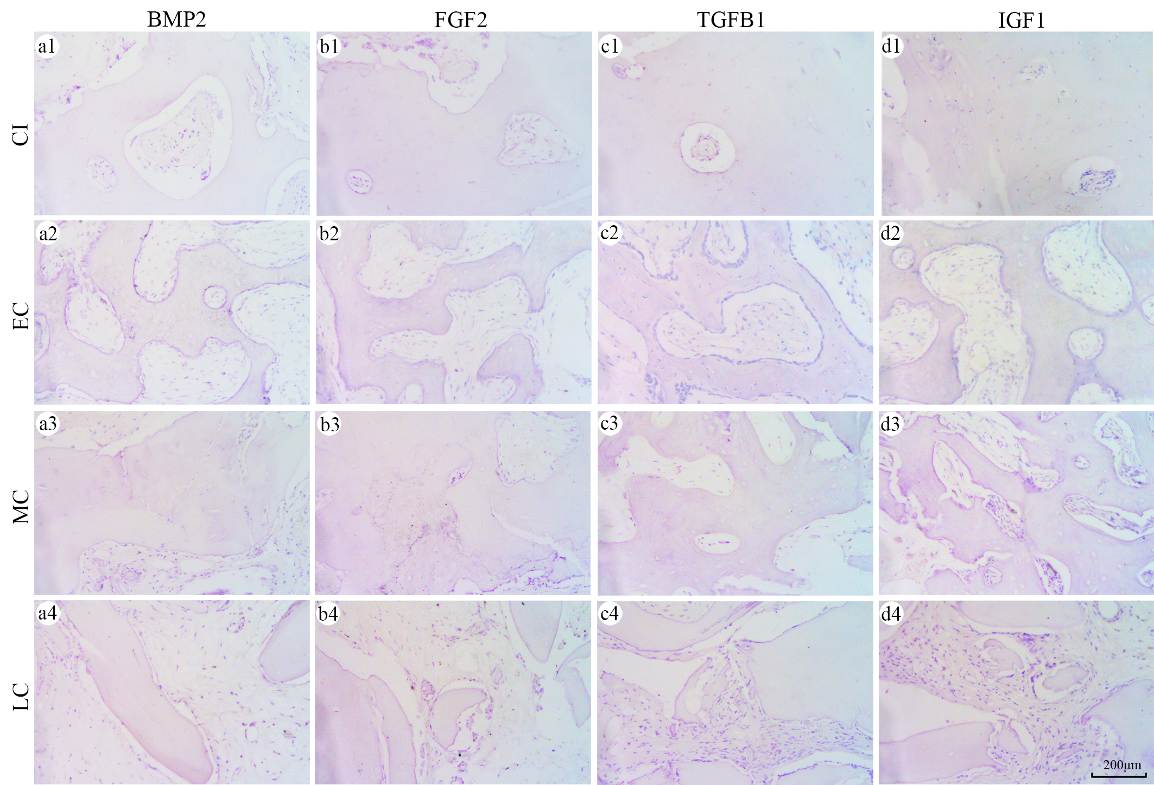


Supplementary Fig. S1. Immunohistochemistry negative control.


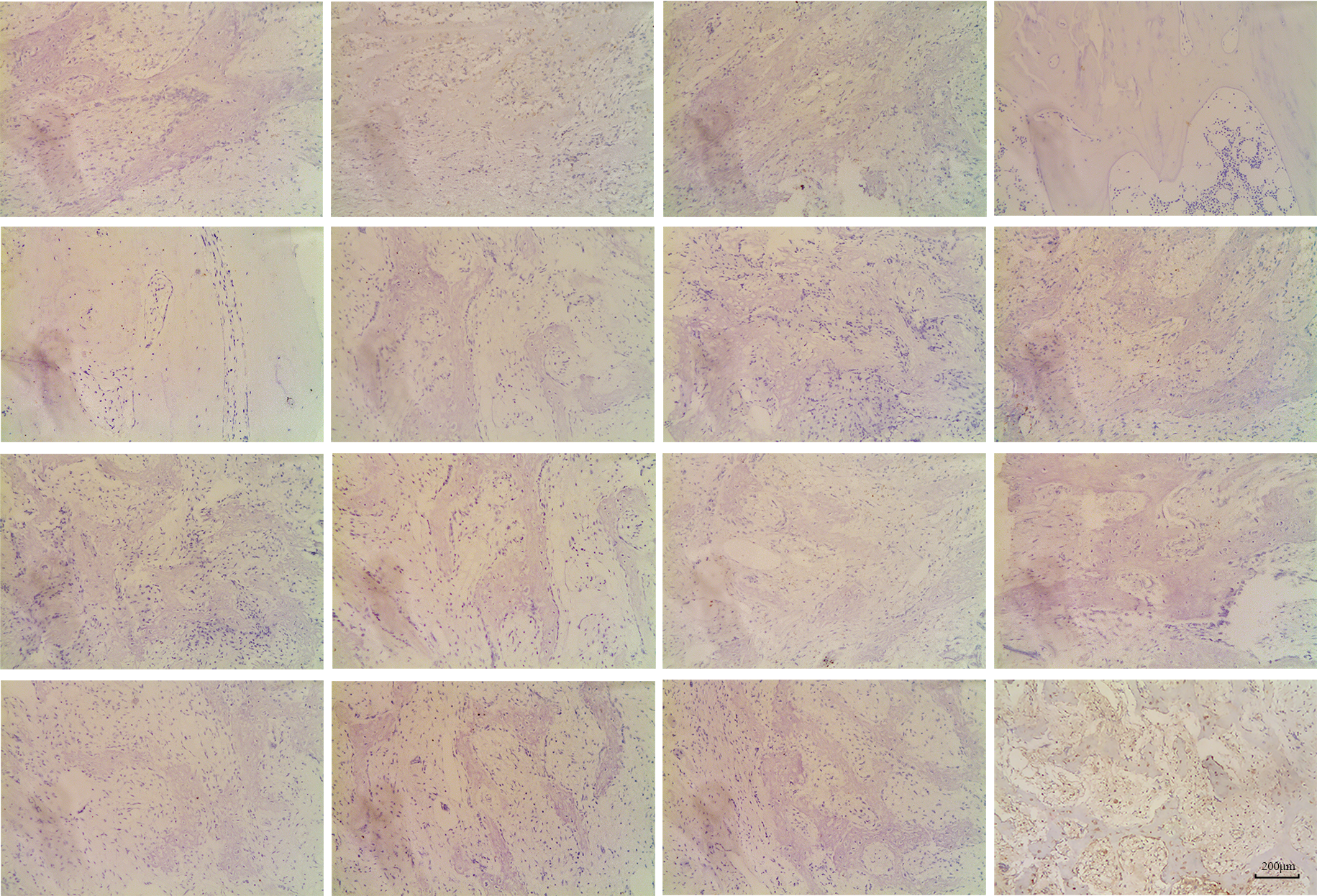


Supplementary Fig. S2. Positive results for TGF-ß1 in the late callus group


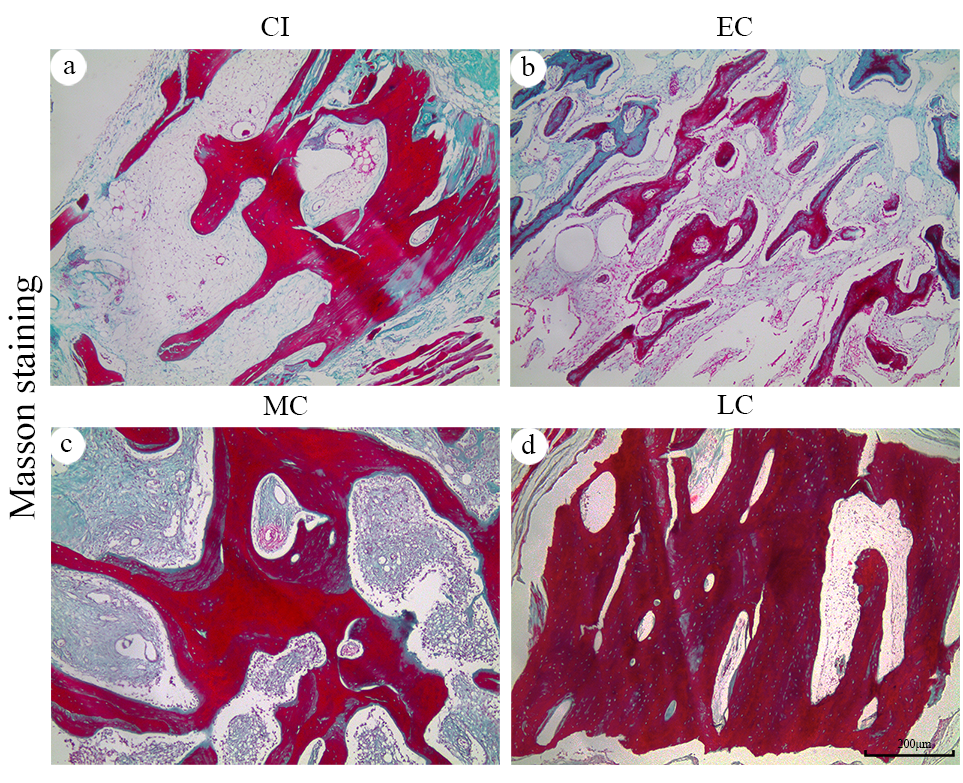


Supplementary Fig. S3. Masson staining for bone callus before implantation*.* Green-stained cartilage collagen component was observed in red-stained mature bone tissue in EC and CI, whereas cartilage was rare in MC and LC.


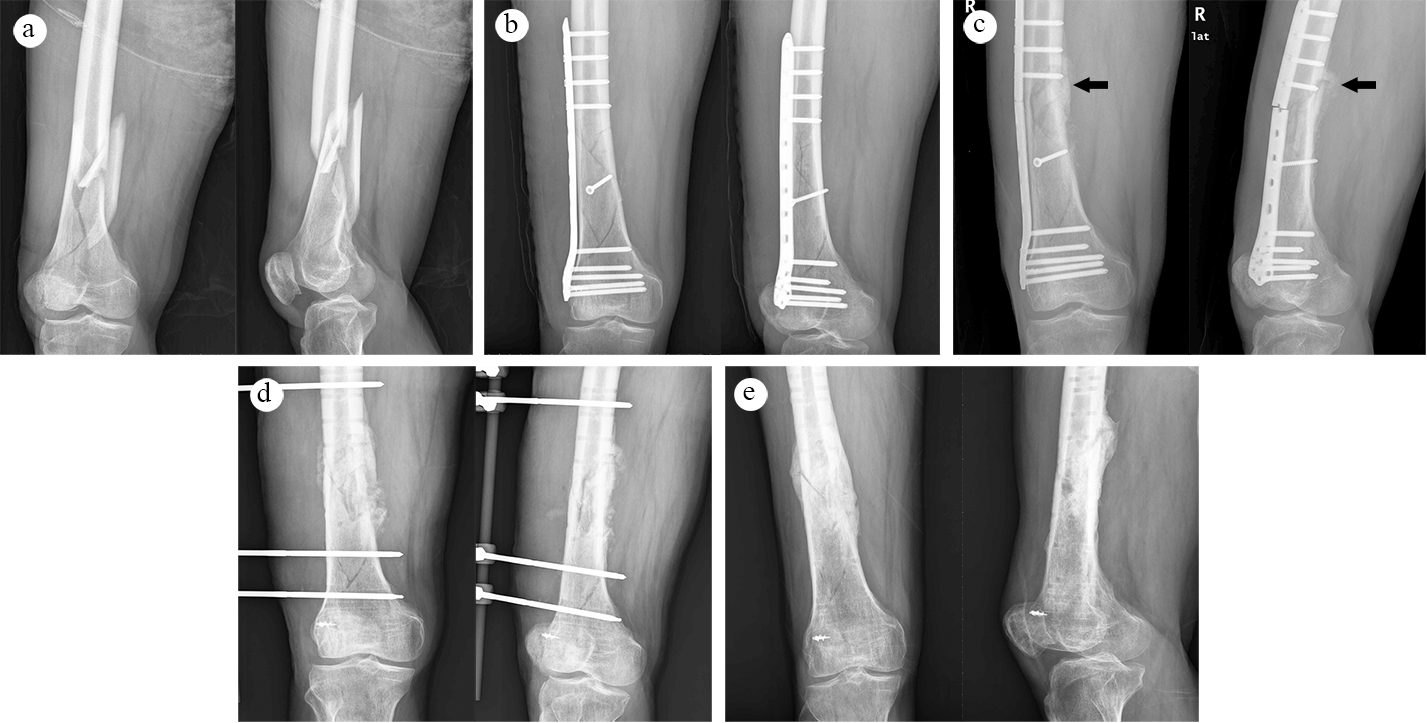


Supplementary Fig. S4. Radiographs of a case with autologous bone callus transplantation in the fracture site. A 66-year-old male with distal fracture of the femur (a); open reduction and plate fixations were applied three days after fracture (b). Six months later, broken plates were found in routine follow-up, and bone callus was formed at the fracture site (black arrows) (c). External fixation was performed, and autologous bone callus mixed with iliac graft was implanted into the bone defect site to promote bone healing (d). Fracture healing was observed one year after the second surgery (e).


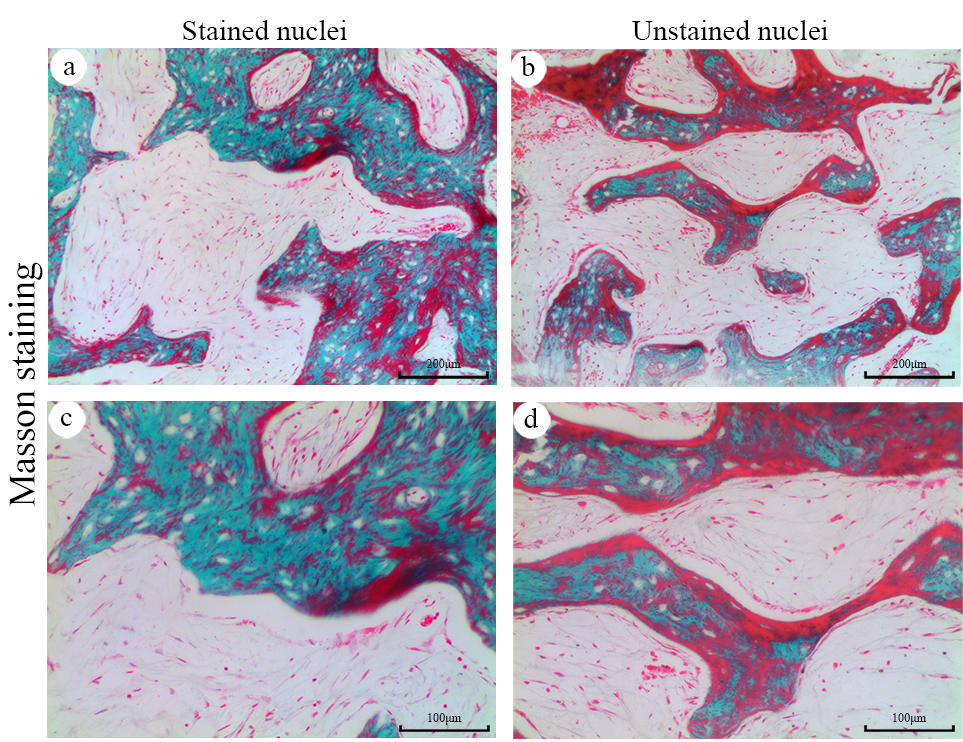


Supplementary Fig. S5. Masson staining for bone callus. Black staining of nuclei by Weigert's hematoxylin was covered by ponceau-acid fuchsin.

Supplementary Table S1. Osteoblasts number of bone callus

| Group | CI | EC | MC | LC |
| --- | --- | --- | --- | --- |
| Osteoblasts number  (/mm) | 253 ± 85 | 375 ± 59^*^ | 152 ± 30^*^ | 80 ± 32^*^ |

^*^Significantly different from the CI group (P < 0.05)

**Supplementary Table S2.** **Histomorphometry data for the osteoconductive structure of bone callus**

| Group | BV/TV  (%) | Tb.N  (N/mm) | Tb.Th  (mm) | Tb.Sp  (mm) |
| --- | --- | --- | --- | --- |
| CI | 58.9 ± 4.5 | 1.34 ± 0.09 | 0.44 ± 0.04 | 0.35 ± 0.02 |
| EC | 43.5 ± 7.7^*^ | 1.44 ± 0.03^*^ | 0.37 ± 0.02^*^ | 0.39 ± 0.02^*^ |
| MC | 70.4 ± 2.6^*^ | 1.23 ± 0.02^*^ | 0.51 ± 0.02^*^ | 0.31 ± 0.01^*^ |
| LC | 75.3± 3.4^*^ | 1.15 ± 0.03^*^ | 0.54 ± 0.02^*^ | 0.21 ± 0.02^*^ |

^*^Significantly different from the CI group (P < 0.05)

Supplementary Table S3. Micro-CT data for the osteoconductive structure of bone callus

| Group | Porosity  (%) | Tb.N (N/mm) | Tb.Th  (mm) | Tb.Sp  (mm) |
| --- | --- | --- | --- | --- |
| CI | 71.5 ± 3.2 | 1.52 ± 0.06 | 0.30 ± 0.06 | 0.24 ± 0.03 |
| EC | 71.2 ± 6.9 | 1.76 ± 0.07^*^ | 0.24 ± 0.02^*^ | 0.26 ± 0.02 |
| MC | 42.8 ± 5.0^*^ | 1.34 ± 0.04^*^ | 0.36 ± 0.03^*^ | 0.16 ± 0.02^*^ |
| LC | 34.4 ± 2.9^*^ | 1.21 ± 0.04^*^ | 0.39 ± 0.04^*^ | 0.10 ± 0.02^*^ |

^*^Significantly different from the CI group (P < 0.05)

Supplementary Table S4. Biomechanics of bone callus

| Group | Ultimate load  (N) | Elastic modulus (MPa) |
| --- | --- | --- |
| CI | 36.4 ± 10.5 | 224 ± 50 |
| EC | 30.6 ± 11.6 | 193 ± 42 |
| MC | 43.8 ± 14.0^*^ | 250 ± 57^*^ |
| LC | 62.8 ± 11.6^*^ | 459 ± 68^*^ |

^*^Significantly different from the CI group (P < 0.05)

Supplementary Table S5. Histomorphometry for bone callus-induced new bone at 8 weeks

| Group | New bone area  /total area (%) | Tb.N (N/mm) | Tb.Th  (μm) | Tb.Sp  (mm) |
| --- | --- | --- | --- | --- |
| CI | 20.0 ± 1.8 | 0.47 ± 0.05 | 12.4 ± 2.37 | 2.13 ± 0.20 |
| EC | 28.3 ± 2.9^*^ | 0.70 ± 0.03^*^ | 15.5 ± 2.44 | 1.41 ± 0.05^*^ |
| MC | 14.5 ± 3.0^*^ | 0.23 ± 0.02^*^ | 14.0± 1.98 | 4.36 ± 0.41^*^ |
| LC | 5.2 ± 0.7^*^ | 0.16± 0.04^*^ | 13.5 ± 2.73 | 6.37± 1.32^*^ |

^*^Significantly different from the CI group (P < 0.05)

Supplementary Table S6. Histomorphometry for bone callus-induced new bone at 12 weeks

| Group | New bone area  /total area (%) | Tb.N (N/mm) | Tb.Th  (μm) | Tb.Sp  (mm) |
| --- | --- | --- | --- | --- |
| CI | 30.8 ± 3.7 | 0.65 ± 0.06 | 18.4 ± 2.32 | 1.53 ± 0.14 |
| EC | 39.5 ± 3.4^*^ | 0.85 ± 0.02^*^ | 18.9 ± 3.21 | 1.16 ± 0.03^*^ |
| MC | 23.5 ± 8.0^*^ | 0.32 ± 0.09^*^ | 17.5± 2.06 | 3.35 ± 0.95^*^ |
| LC | 9.6 ± 1.3^*^ | 0.26± 0.05^*^ | 17.5± 3.34 | 3.95± 0.76^*^ |

^*^Significantly different from the CI group (P < 0.05)

Supplementary Table S7. Micro-CT for bone callus-induced new bone at 8 weeks

| Group | ΔBV/TV  (%) | ΔTb.N (N/mm) | ΔTb.Th  (μm) | ΔTb.Sp  (μm) |
| --- | --- | --- | --- | --- |
| CI | 7.2 ± 1.1 | 0.34 ± 0.09 | 50.1 ± 10.3 | 68.3 ± 10.3 |
| EC | 11.4 ± 1.4^*^ | 0.58 ± 0.12^*^ | 60.2 ± 12.4 | 99.5 ± 11.4^*^ |
| MC | 4.6 ± 0.7^*^ | 0.14 ± 0.05^*^ | 54.5 ± 7.1 | 51.6 ± 8.4^*^ |
| LC | 2.3 ± 0.5^*^ | 0.08 ± 0.01^*^ | 36.3 ± 8.6 | 34.3 ± 7.6^*^ |

^*^Significantly different from the CI group (P < 0.05)

Supplementary Table S8. Micro-CT data for bone callus-induced new bone at 12 weeks

| Group | ΔBV/TV  (%) | ΔTb.N (N/mm) | ΔTb.Th  (μm) | ΔTb.Sp  (μm) |
| --- | --- | --- | --- | --- |
| CI | 12.4 ± 1.5 | 0.50 ± 0.11 | 73.4 ± 11.2 | 134 ± 18 |
| EC | 17.8 ± 2.3^*^ | 0.79 ± 0.13^*^ | 78.3 ± 13.3 | 164 ± 25^*^ |
| MC | 8.3 ± 0.8^*^ | 0.21 ± 0.09^*^ | 67.1 ± 8.4 | 93.2 ± 16.9^*^ |
| LC | 6.5 ± 0.7^*^ | 0.16 ± 0.03^*^ | 63.4 ± 14.1 | 57.6 ± 13.8^*^ |

^*^Significantly different from the CI group (P < 0.05)
